# Racial, Gender, and Size Bias in a Medical Graphical Abstract Gallery: A Content Analysis

**DOI:** 10.1089/heq.2023.0026

**Published:** 2023-09-27

**Authors:** Jessica P. Cerdeña, Jennifer W. Tsai, Chloe Warpinski, Robert F. Rosencrans, Clarence C. Gravlee

**Affiliations:** ^1^ Department of Family Medicine, Middlesex Health, Middletown, Connecticut, USA.; ^2^ Institute for Collaboration on Health, Implementation, and Policy (InCHIP), University of Connecticut, Storrs, Connecticut, USA.; ^3^ Department of Anthropology, University of Connecticut, Storrs, Connecticut, USA.; ^4^ Department of Emergency Medicine, Yale School of Medicine, New Haven, Connecticut, USA.; ^5^ Department of Anthropology, University of Florida, Gainesville, Florida, USA.; ^6^ MD-PhD Training Program, University of Florida College of Medicine, Gainesville, Florida, USA.; ^7^ Medical Scientist Training Program, University of Alabama at Birmingham, Birmingham, Alabama, USA.

**Keywords:** race, gender, size bias, social determinants of health, graphical abstracts, science communication

## Abstract

**Introduction:**

Graphical abstracts may enhance dissemination of scientific and medical research but are also prone to reductionism and bias. We conducted a systematic content analysis of the *Journal of Internal Medicine (JIM) Graphical Abstract Gallery* to assess for evidence of bias.

**Materials and Methods:**

We analyzed 140 graphical abstracts published by *JIM* between February 2019 and May 2020. Using a combination of inductive and deductive approaches, we developed a set of codes and code definitions for thematic, mixed-methods analysis.

**Results:**

We found that *JIM* graphical abstracts disproportionately emphasized male (59.5%) and light-skinned (91.3%) bodies, stigmatized large body size, and overstated genetic and behavioral causes of disease, even relative to the articles they purportedly represented. Whereas 50.7% of the graphical surface area was coded as representing genetic factors, just 0.4% represented the social environment.

**Discussion:**

Our analysis suggests evidence of bias and reductionism promoting normative white male bodies, linking large bodies with disease and death, conflating race with genetics, and overrepresenting genes while underrepresenting the environment as a driver of health and illness. These findings suggest that uncritical use of graphical abstracts may distort rather than enhance our understanding of disease; harm patients who are minoritized by race, gender, or body size; and direct attention away from dismantling the structural barriers to health equity.

**Conclusion:**

We recommend that journals develop standards for mitigating bias in the publication of graphical abstracts that (1) ensure diverse skin tone and gender representation, (2) mitigate weight bias, (3) avoid racial or ethnic essentialism, and (4) attend to sociostructural contributors to disease.

## Introduction

Graphical abstracts are an increasingly popular form of disseminating scientific and medical research, with some publications requiring graphical abstracts for selected submissions.^1–3^ Graphical abstracts increase exposure for journals and authors, facilitate sharing of publications on digital platforms including social media, boost article-level metrics, and may speed the translation of new information to clinical practice.^4–7^ Previous educational scholarship also suggests that multimodal information improves learning.^3,8,9^ These effects allow deeper engagement, retention, and recall of information that may improve application of new scientific data.^1,3,10,11^

However, the characteristics that make graphical abstracts useful dissemination tools also create potential pitfalls by oversimplifying results, focusing on positive findings, or flattening conceptual nuances that limit accuracy.^1,4,12^ Furthermore, graphical abstracts, such as all forms of scientific translation and representation, illuminate many tacit cultural assumptions in science and society, modulating its communication and comprehension.^13^

A recent, high-profile example illustrates these pitfalls. In May 2020, the *Journal of Internal Medicine (JIM)* published a review article by B. A. Gower and L. A. Fowler originally titled “Obesity in African Americans: Is physiology to blame?”^14^ The accompanying graphical abstract, produced by the journal rather than the authors, depicted a black woman in a blue sweater surrounded by three images: (1) an insulin molecule bound to a membrane receptor labeled “Inuslin [*sic*] sensitivity”; (2) a honey pot and dipper, soda can, and cupcake labeled “Diet glycemic load”; and (3) a liver, pancreas, and duodenum section labeled “Insulin secretion and clearance.” The illustration of the woman closely resembles a still from the 2009 movie *Precious*.^15^ The graphical abstract received widespread online backlash, while tracking in the top 5% of research output scored by Altmetric.^16–19^

In response, the publisher removed the graphical abstract, and the journal apologized for having disseminated an image that “perpetuated racial insensitivities and negative weight bias and stigma associated with the disease of obesity.”^20^

Our study grew out of concern that the issues identified in the retracted abstract may often remain undetected. To pursue this inquiry, we conducted a qualitative content analysis of the graphical abstracts published in *JIM*. We studied representations of human bodies and disease to probe for issues of reductionism or bias. We focused on the representation of human figures and the extent to which explanatory models of disease depicted in the graphical abstract relied on genetic versus social or environmental factors. To our knowledge, this is the first systematic content analysis of published graphical abstracts.

## Materials and Methods

### Data

*JIM* is an influential medical journal that currently ranks 12th among the 169 journals in the General and Internal Medicine category and was the highest ranking journal with a graphical abstract gallery at the time of our analysis.^21^ We examined all graphical abstracts published between February 2019, when the series began, and May 2020, when controversy around the Gower and Fowler article erupted (*N*=140). We downloaded all available images from the *JIM Graphical Abstract Gallery*,^22^ along with bibliographic details (RIS format) and the corresponding article. We assembled this data set in MAXQDA 2020.^23^

### Codebook development and coding

We developed a set of codes and code definitions for thematic analysis, using a combination of inductive and deductive techniques for identifying themes.^24^ Two coders (J.P.C. and J.W.T.) annotated elements independently and developed codes. J.P.C. developed 171 preliminary inductive codes and J.W.T. began with 24 broader deductive codes. As a team, we compared codes from the initial review and settled on 52 codes organized into six major themes. The codebook is available in the Supplementary Data.

J.P.C. and J.W.T. then applied the codebook independently to the full sample. Each code was applied only to relevant segments of an image, such that coding required two decisions: (1) how to segment the image and (2) which code(s) to apply to each segment. On average, coders agreed 75% of the time across codes (range 40–100%). Through discussion, we refined the codebook to clarify definitions, and a third coder (C.C.G.) resolved discrepancies to prepare the final data set.

### Analysis

We adopted a mixed-methods approach to analysis. To summarize the occurrence of themes across the data set, we examined frequency distributions of codes by graphical abstracts, coded segments, and area of images. We used exploratory visual tools in MAXQDA, including multidimensional scaling and hierarchical cluster analysis,^25^ to identify patterns in the co-occurrence of codes. Throughout the process, we wrote memos to record questions, observations, and interpretations of the graphical abstracts.

## Results

The studies included in our analysis largely presented results from clinical and translational science research across varied specialties, including cardiology, neurology, endocrinology, gastroenterology, and psychiatry. Many reported data on disease associations identified through clinical registries or results from hospital-based trials, whereas others reported more laboratory-based data interrogating pathogenic mechanisms.

### Representations of bodies and racialization

Table 1 presents the frequencies and coverage (measured as image area) of codes corresponding to people and populations. At least one of these codes occurred in 96 graphical abstracts (68.6% of the total sample), and together they were applied a total of 435 times. Comparisons across abstracts and coded segments reveal a similar pattern. Figures were more commonly coded as silhouettes, male, and light-skinned than they were as illustrations, female, and dark-skinned.

**Table 1.** Frequency and Area Coverage of Codes Related to People and Populations, by Graphical Abstracts (*N*=140) and Coded Segments (*N*=435)

Graphical abstracts					Coded segments				
Rank order	Code	Frequency	Percentage	% of entire image area	Rank order	Code	Frequency	Percentage	% of coded area
1	Silhouette	52	37.1	9.2	1	Silhouette	89	20.5	41.5
2	Male	50	35.7	9.1	2	Male	72	16.6	41.0
3	Illustration	32	22.9	7.9	3	Female	49	11.3	23.4
4	Light skin	32	22.9	6.9	4	Light skin	42	9.7	31.1
5	Female	31	22.1	5.2	5	Illustration	38	8.7	35.7
6	Large body size	19	13.6	2.0	6	Large body size	27	6.2	8.9
7	Origin	16	11.4	1.2	7	Origin	23	5.3	5.2
8	Map	12	8.6	3.0	8	Youth	19	4.4	1.7
9	Aging	10	7.1	1.2	9	Aging	17	3.9	5.6
10	Body size	10	7.1	0.4	10	Racial or ethnic category	13	3.0	0.1
11	Youth	9	6.4	0.4	11	Body size	12	2.8	1.9
12	Population	7	5.0	0.3	12	Map	12	2.8	13.6
13	Intrauterine	5	3.6	0.4	13	Population	8	1.8	1.3
14	Racial or ethnic category	4	2.9	0.0	14	Intrauterine	5	1.2	1.8
15	Demographic	4	2.9	0.3	15	Demographic	5	1.2	1.3
16	Dark skin	4	2.9	1.4	16	Dark skin	4	0.9	6.4
	Abstracts with code(s)	96	68.6	77.7		Total	435	100	
	Abstracts without code(s)	44	31.4	22.3					
	Analyzed abstracts	140	100.0	100.0					

Of the abstracts coded for sex, 70.4% contained codes for male and 43.7% codes for female. Where sex of individual bodies could be determined, 59.5% of bodies were coded as male and 40.5% were coded as female. Considering only segments of images coded for people or populations, male bodies accounted for 41.0% of the coded area and female bodies for 23.4%.

Twenty abstracts (14.3%) contained some mention of body size. Of those, 95.0% referenced large body size and 50.0% referenced body size more generally. Case 1 demonstrates how the use of a silhouette emphasizes a man's large body size and individual behaviors, despite inattention to body mass and behavior in the article text.

**CASE 1.**
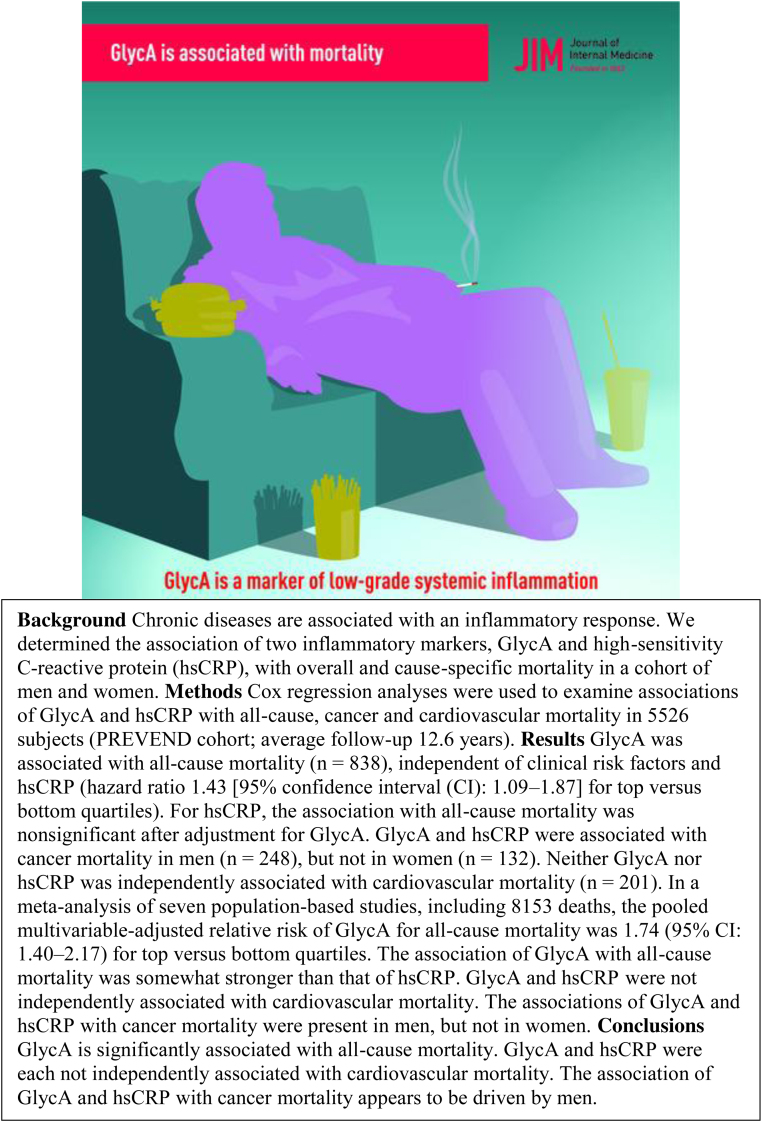
Gruppen and colleagues^27^ describe results from a study and meta-analysis examining the association of two inflammatory biomarkers—GlycA and hsCRP—with overall and cause-specific mortality. They found that GlycA is significantly associated with all-cause mortality and that an identified association of GlycA and hsCRP with cancer mortality appears to be driven by men. The abstract for the study shows a large-bodied man, colored in bright purple, slouching in a sofa chair, smoking a cigarette. Although his facial features are absent, fat accumulations in his chest, abdomen, and suprapubic area are highlighted. To the man's right, on the arm of the sofa, is a hamburger, colored in yellow, and at his feet are a soda with a straw and a container of French fries, also colored in yellow. The posture of the male figure suggests fatigue, even laziness or inactivity; however, the authors did not consider physical activity in their analyses. The man's body is also large, implying obesity, yet the effect of BMI is only briefly mentioned, suggesting that BMI increased at higher GlycA levels (p. 599). In addition, the figure is holding a cigarette even though smoking status was either a control variable or not mentioned in all of the studies included in the article. Finally, the graphical abstract suggests that diet is an important contributor to the author's findings by emphasizing the hamburger, soda, and French fries, yet “diet” is only mentioned once in the article. These artistic decisions have the effect of suggesting behavioral contributions to increased mortality when, in fact, the authors only noted an association with inflammatory biomarkers. The depiction of a large-bodied person as indolent, surrounded by fast food, reinforces weight bias by suggesting this man is responsible for his own imminent mortality. BMI, body mass index; hsCRP, high-sensitivity C-reactive protein.

Just 5.0% of abstracts referenced populations rather than individuals: all of these represented populations to convey disease risk or study samples. Human figures in representations of populations frequently resembled silhouetted icons used to denote men's and women's restrooms. In two instances, the icons appeared deliberately genderless. In all but one abstract, the population figures were colored in nonskin tones; in Naucler et al., the figures are mostly rendered with light-skin phenotypes.^26^

Only 18.8% of abstracts referenced non-European or American countries of origin, including South Africa, China, and India. Sweden and the United Kingdom were mentioned most often, each appearing in four abstracts. Similarly, of nine regional maps, only one included areas outside of Europe or North America.

Racial or ethnic categories were mentioned in just four abstracts (2.9%), with varied terminology. Across abstracts, both “African American” and “black” are used as well as “white” and “Caucasian.” In addition, both “Mexican” and “Hispanic” are included independently in the graphical abstract for Le et al.^28^ With respect to skin phenotype, 94.1% of abstracts that depicted bodies contained light-skinned bodies whereas only 11.8% contained dark-skinned bodies. Of the bodies coded with skin phenotypes, 91.3% were classified as light and just 8.7% were classified as dark. Light skin accounted for almost 10% of the coded area, while dark skin was less than 1%. There was no significant difference in the representation of light- or dark-skin phenotypes by gender or body size.

### Representations of disease

Table 2 presents the frequency and coverage (measured as image area) of codes corresponding to genetic, behavioral, and environmental contributors to disease. At least one of these codes occurred in 57 graphical abstracts (40.7% of the total sample), and together they were applied a total of 170 times. Both the frequency across abstracts and the frequency across segments reveal a similar pattern.

**Table 2.** Frequency and Area Coverage of Codes for Contributors to Disease Risk, by Graphical Abstracts (*N*=140) and Coded Segments (*N*=170)

Graphical abstracts					Coded segments				
Rank order	Code	Frequency	Percentage	% of entire image	Rank order	Code	Frequency	Percentage	% of coded area
1	Genetics	29	20.7	3.4	1	Genetics	44	25.9	50.7
2	Diet	16	11.4	1.2	2	Diet	32	18.8	17.6
3	Physical environment	8	5.7	0.3	3	Epigenetics	15	8.8	5.7
4	Physical activity	8	5.7	1.1	4	Physical activity	14	8.2	15.9
5	Epigenetics	8	5.7	0.2	5	Physical environment	14	8.2	4.2
6	Sedentary	7	5.0	0.5	6	Lifestyle	11	6.5	1.7
7	Lifestyle	7	5.0	0.1	7	Sedentary	10	5.9	7.5
8	Smoking	6	4.3	0.1	8	Fruits and vegetables	8	4.7	9.5
9	Fruits and vegetables	5	3.6	0.6	9	Social environment	7	4.1	0.4
10	Social environment	4	2.9	0.0	10	Smoking	6	3.5	1.5
11	Fast food	3	2.1	0.1	11	Fast food	6	3.5	1.3
12	Alcohol	3	2.1	0.0	12	Alcohol	3	1.8	0.3
	Abstracts with code(s)	57	40.7	6.8		Total	170	100	
	Abstracts without code(s)	83	59.3	93.2					
	Analyzed Abstracts	140	100.0	100.0					

References to genetic factors are the most common theme. The 44 segments (in 29 abstracts) coded as “genetics” include a mixture of textual and visual elements. Visual representations of genetic factors are evident in more than half (50.7%) of the image area coded for contributors to disease risk. By far the most common element is the double helix occurring in nearly two-thirds (19 of 29) of the graphical abstracts that reference genetic factors. The double helix often invokes abstract ideas about heredity or genetic predisposition to disease, even when that is not the focus of the article it references. Case 2 links DNA helices with ethnic predisposition to diabetes.^29^

**CASE 2.**
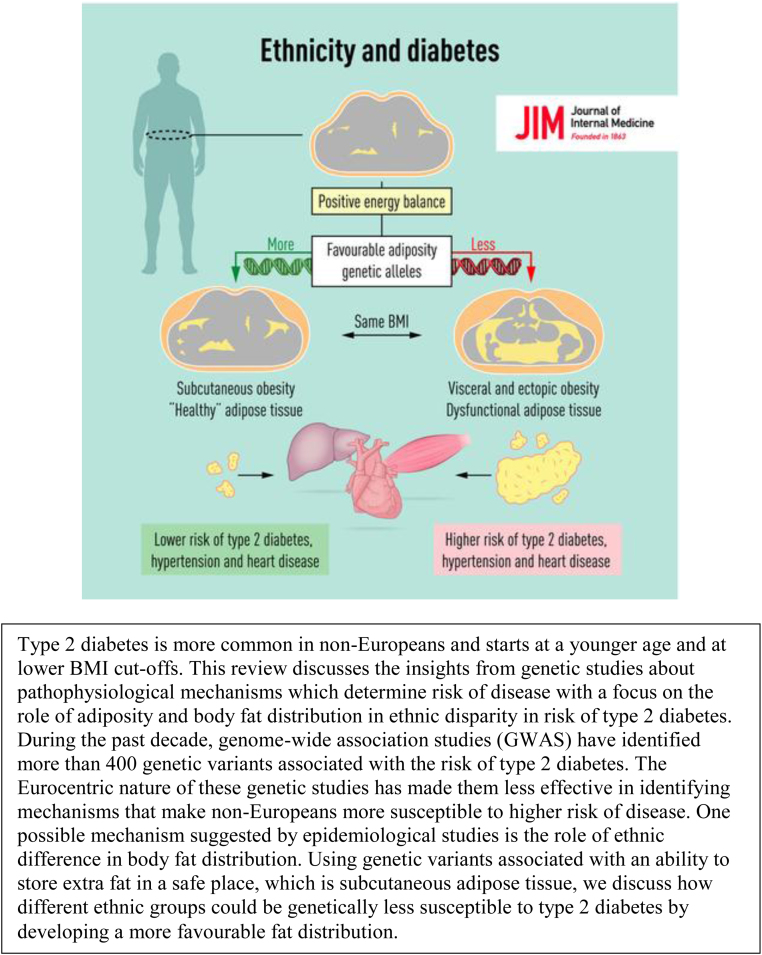
Yaghootkar et al.^29^ review the genetics literature on “ethnic differences” in adiposity and risk for type 2 diabetes. Their discussion assesses the potential contributor of ethnic variation in genetic variants associated with increased fat storage to explain their presumption that “non-Europeans” are more susceptible to diabetes relative to Europeans. The graphical abstract accompanying Yaghootkar et al.^29^ illustrates two problems surrounding conceptions of ethnicity: First, the article and the graphical abstract use ethnicity as a proxy for unspecified risk factors; second, the authors assume that small quantities of human genetic variation are distributed in an ethnically discontinuous manner and correspond to differences in the risk for disease. The focus of the abstract, as the headline notes, is “ethnicity and diabetes.” Ethnicity is not defined, and none of the complexities of that concept is captured in the graphical abstract, leaving readers to fill in the gaps with whatever assumptions they make about ethnicity and its relationship to disease. If readers are prone to interpret ethnic differences in health as a result of genetic variation, they will find encouragement for that view in the graphical abstract. The abstract depicts a process model that begins with “positive energy balance” and ends with either a lower or higher risk for cardiometabolic disease. Risk is simplified as a binary outcome, and the main determinant is whether people have more or fewer “favorable adiposity genetic alleles.” Genetic variation is color-coded into two categories, with a green double helix resulting in “healthy” adipose tissue and a red one leading to “dysfunctional” adipose tissue. Because no other influences on adiposity or cardiometabolic risk are depicted, the implication is that genetic variation is the key to ethnic differences in diabetes. Moreover, the representation of genetic variation as two color-coded double helixes promotes categorical thinking and implies that some alleles are intrinsically maladaptive, while others promote good health. The featuring of the double helix further obscures the environmental interactions with genetics that together engender disease risk.

The graphical abstracts portray a relatively small set of nongenetic contributors to disease risk (Table 2). Diet is most prominent, accounting for 18% of the image area coded for disease risk factors. When graphical abstracts include physical activity it is a prominent theme: physical activity represents only 8.2% of the coded segments, but 15.9% of the image area coded for disease risk factors. Images indicating sedentary behavior also act as prominent visual elements evident in 5.9% of coded segments but 7.5% of image area.

All 11 segments coded as “lifestyle” refer to textual elements, including five that explicitly reference “lifestyle” and five that refer to sleep patterns. The social environment is also represented only by textual references such as “psychosocial,” “high income,” and “stress.” Likewise, the physical environment is represented primarily by textual references such as “geo-physical environment” and “UV-light.” The use of textual rather than visual elements means that the area of graphical abstracts devoted to physical environment, lifestyle, and social environment is low, relative to their frequency. Case 3 illustrates how lifestyle and environmental contributors to health and disease are represented in limited ways in favor of genetic motifs,^30^ even when environmental factors are central to the disease processes in question.

**CASE 3.**
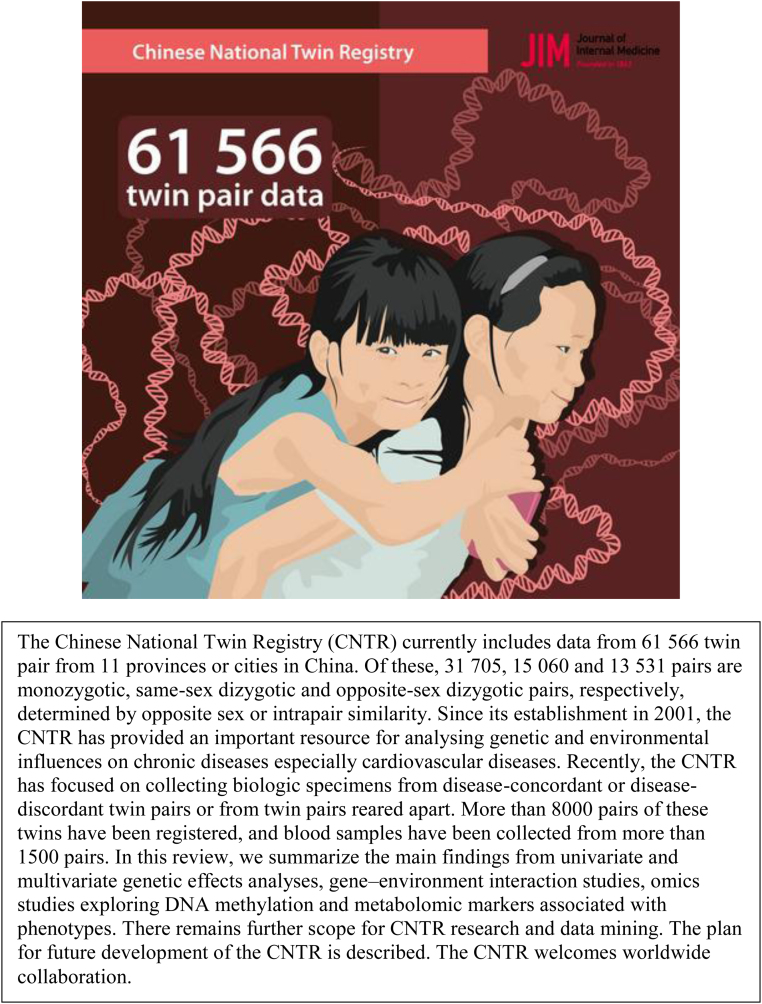
In the article itself, Gao et al.^30^ describe the CNTR, which “aimed to study the genetic and environmental contributions to complex diseases, with particular emphasis on cardiovascular diseases” (p. 300). An important design aspect of the CNTR is its attention to nongenetic (behavioral and environmental) factors to identify what Gao et al.^30^ describe as “lifestyle-discordant and concordant twin pairs” (p. 303). Among the lifestyle variables available for analysis are smoking, alcohol consumption, fruit and vegetable consumption, and physical activity (p. 302). The registry also includes a range of clinical and anthropometric measures (e.g., height, weight, waist and hip circumferences, blood pressure) and standard sociodemographic measures such as marital status and educational attainment (p. 304). In short, Gao et al.^30^ describe a fairly broad range of nongenetic influences on cardiovascular disease, and the science they summarize in the article conveys the importance of environmental modifiers of disease risk. Yet the graphical abstract gives a different impression. It includes only two visual elements: an illustration of twin sisters against a background of swirling double helices of DNA. None of the nongenetic contributors to disease described in the article or available in the registry itself is represented in the graphical abstract. CNTR, Chinese National Twin Registry.

**FIG. 1.**
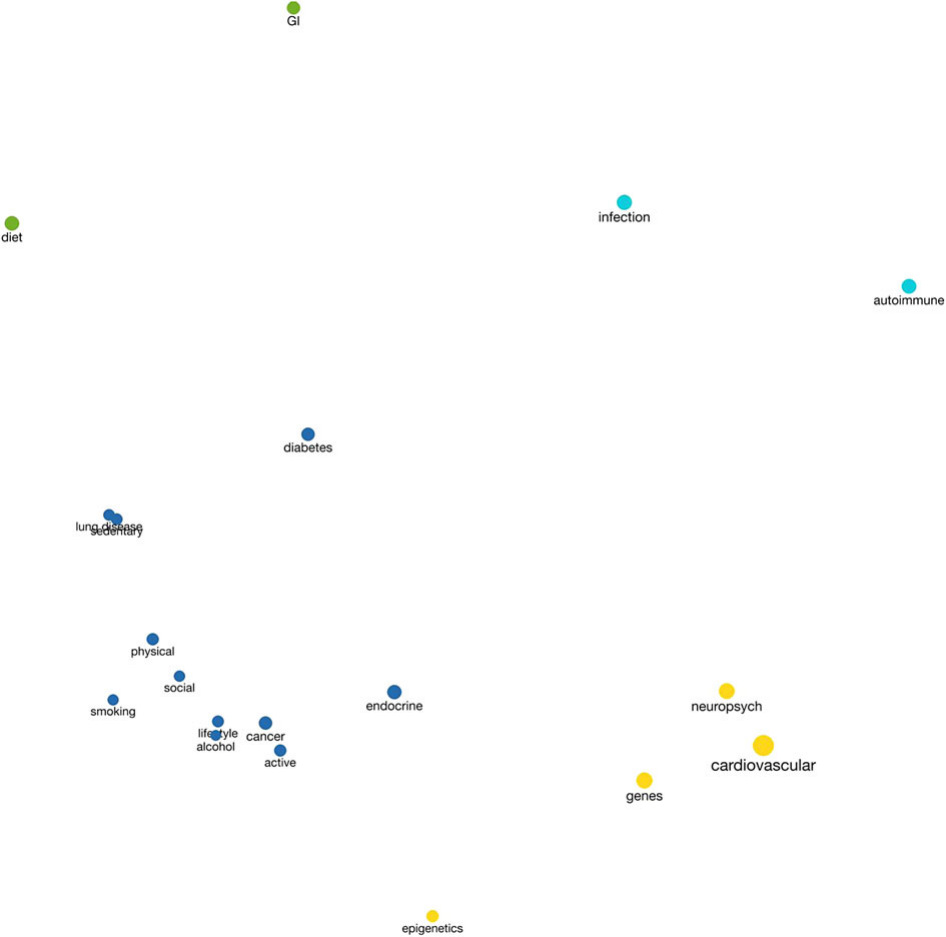
Multidimensional scaling plot of codes related to disease risk. A multidimensional scaling plot demonstrating the co-occurrence of codes in two-dimensional space. Four predominant clusters appear: The *blue* cluster features disease processes, including diabetes, cancer, and endocrine disorders along with major lifestyle contributors such as physical activity and smoking. The *yellow* cluster includes cardiovascular and neuropsychiatric diseases along with genes and epigenetics. The *aqua* cluster groups infections and autoimmune disorders and the *green* cluster groups gastrointestinal disease and diet.

Figure 1 visualizes the co-occurrence of contributors to disease and types of pathology in the graphical abstracts. The largest cluster includes four types of pathology (e.g., cancer, diabetes) and seven contributors to disease risk (e.g., sedentary behavior, physical environment). The next largest cluster includes cardiovascular and neuropsychiatric pathology as well as genetic and epigenetic contributors to disease. The remaining two clusters are formed by diet and gastrointestinal pathology and by infection and autoimmune disease. The first dimension, from lower-left to upper-right, ranges from chronic to infectious disease. The second dimension, ranging from upper-left to lower-right, appears to distinguish between external and internal processes. External influences such as diet, physical activity, and infection fall on one side of the diagonal; internal ones such as genetic variation, epigenetic regulation, and autoimmune responses appear on the other. Codes that appear on the edges of clusters illustrate how these dimensions intersect (e.g., diabetes co-occurs with codes in the adjacent clusters of diet, genes, and infection).

## Discussion

Our analysis found that graphical abstracts in *JIM* strongly emphasize male, light-skinned bodies from European and North American countries; commonly include negative representations of large body size; and overstate genetic and behavioral causes of disease while minimizing environmental ones. These findings suggest that despite the rigorous peer review process typical of medical journals such as *JIM*, accompanying graphical abstracts exhibit cultural biases and reductionist models of disease that have long been targets of criticism. These biases undermine the validity of scientific communication and may contribute to lower quality of care for certain marginalized patients.^31–35^

Our analysis identified a disproportionate representation of male bodies. According to the Global Change Data Lab, as of 2017, 49.6% of the global population was female.^36^ However, only 31 graphical abstracts in our sample depicted female bodies compared with 50 that represented male bodies, and just 40.5% of the bodies represented in these graphical abstracts were coded as female. This imbalance may reflect and reinforces a broader cultural model of the default human subject as male and white.^37,38^ In the graphical abstracts we examined, normative expressions of physiology or pathology are illustrated disproportionately with light skin, well-defined musculature, thin abdomens, and breastless torsos. Gender biases exist in the diagnosis of many illnesses, for example, acute myocardial infarction, which commonly presents with unique symptoms or more complex combinations of symptoms in women.^38–42^

Causally linking such disparities to gender biases in representation is challenging; however, it is notable that even the American Heart Association's Common Heart Attack Warning Signs^43^ graphic displays a genderless figure with the classic sign of chest pain listed next to the number one, to signify its primary role in diagnosis. Such graphics constitute an important component of illness scripts, which help shape providers' knowledge and diagnostic frameworks. Even seemingly genderless representation could produce inequitable outcomes by suggesting that classic symptoms transcend gender. Gender signifiers in visual communication must be assessed critically with a continuous posture of reflexivity to reduce gender-based health inequities.^44^

We further observed overrepresentation of European and North American countries relative to other regions of the world. This disparity is further reflected by overrepresentation of light-skin phenotypes. These patterns reflect the inverse of the global population: most people in the world have dark skin,^45^ yet only a minority of images in these abstracts represented darker skin phenotypes. Underrepresentation of darker skin in visual summaries of medical research contributes to existing gaps in medical education materials, which may play a role in the increased morbidity and mortality for darker skinned patients.^46–49^

Explicit references to race and ethnicity were not common, but when they occurred, we observed problematic terminology. Both “Mexican” and “Hispanic” were used as undefined and independent groups in a study that took place in the United States, where these groups are not mutually exclusive. This imprecision reflects long-standing inconsistencies in the use of racial categories in biomedical publications.^50^

Abstracts in our sample disproportionately reference large body size, often linking it to inactivity, disease, and death. Despite causal claims about the health consequences of obesity, existing data suggest that the highest risk for mortality occurs at the extreme ends of the weight spectrum (body mass index [BMIs] <18.5 and >35), and even this may vary with people's experiences of racialization.^51,52^ Evidence suggests that negative attitudes held by health care providers toward large-bodied patients may unjustly impact the care they receive.^53^ Weight discrimination is associated with adverse health effects, including increased allostatic load and immune and cardiovascular biomarkers such as C-reactive protein and resting heart rate.^54,55^ Weight discrimination in graphical abstracts risks perpetuating these harms.^17^

Our analysis demonstrates that *JIM'*s graphical abstracts overwhelmingly attribute disease to genetic causes. Although genetic factors contribute, they are not the primary cause of common chronic disease.^56^ More than half of all the graphical abstracts depicting disease risk referred to genetic factors, and nearly two-thirds of these abstracts featured illustrations of DNA—by far the most common visual reference to causes of disease. This suggests that graphical abstracts, which are effective precisely because of their imagery, can reproduce unverified conclusions about the power and influence of DNA.^57^

The disproportionate representation of light-skinned bodies, uncritical and imprecise use of racial and ethnic descriptors, and overstatement of genetic drivers of disease uphold false notions of racial essentialism. Race is a notoriously complex concept; its depiction, use, and representation as a research variable have long been controversial.^58–61^ Scholarly literature establishes race as a dynamic social construct rather than an essential physiological variable, but biomedical research continues to operationalize racial categories and signifiers as innate, genetic characteristics.^62–64^ This treatment of race assumes that racial labels correspond to innate genetic markers and can be used as a proxy for unspecified risk factors that predispose individuals to disease. This assumption is not only inaccurate, but harmful. Significant literature demonstrates that exposure to scientific reports, science education, and even public service announcements that invoke notions of racial essentialism can increase prejudice, racial animosity, and apathy toward inequality.^65–68^

We also found that graphical abstracts in *JIM* represent the environment in limited ways. References to the social environment were uncommon, appearing in only four graphical abstracts. As Case 3 shows,^30^ even when nongenetic variables are analyzed, the graphical abstracts neglect environmental factors as isolated textual elements in favor of expansive swirling DNA helices. The result is that genetic factors were represented in half of the image area related to disease risk factors, while the area allotted to the social environment was only 0.4% of the coded area across all abstracts. This pattern is significant because the promise of graphical abstracts as a genre of science communication lies in the ability to convey complex ideas in a visual form. If social factors are represented as text while the double helix predominates as a visual motif, then graphical abstracts may distort rather than enhance our understanding of disease risk.

Following genetic attributions, the most common risk factors depicted in the analyzed graphical abstracts are individual-level behaviors, particularly diet and physical activity. The focus on individual-level, “lifestyle” factors mirrors the focus of articles published in *JIM*, which reflect widespread assumptions about culpable behavior and disease. This message overlooks social determinants of health that impact access to fresh food and safe exercise spaces and is consistent with neoliberal market structures that emphasize individual responsibility for health and illness—including imperatives to eat well, exercise regularly, and avoid unhealthful behaviors such as smoking and unprotected sex—without consideration of social inequities that limit individual choice.^69^

These assumptions are also evident in Figure 1, which visualizes the co-occurrence of disease conditions and purported genetic and behavioral risk factors in our sample of graphical abstracts. The figure distinguishes chronic degenerative conditions from communicable and autoimmune diseases, as well as risk factors that are external to the body (e.g., diet, sedentary lifestyle) from ones that are internal to the body (e.g., genetics, epigenetics). This pattern suggests a degree of individual responsibility for conditions such as diabetes and cancer and a focus on hereditary components of cardiovascular and neuropsychiatric disease—despite complex etiologies involving genetic and social factors in all these conditions.^70^ Holistic views of illness and health that consider social, biological, environmental, and psychological interactions are necessary for clinicians to develop nuanced assessments of patients and to attend to political–economic and social systems that affect health and care.^71–74^

Although environmental nuances may be more difficult to render visually relative to molecular structures, graphical abstracts have the potential to capture complex dimensions of the sociostructural environment. For instance, in the example of diabetes, trees and a park might represent neighborhood walkability, dilapidated buildings might represent the effect of neighborhood blight on the ability to exercise, and migrant crossing the border crossing might highlight the challenges faced by immigrants and refugees.^75,76^ However, when the environment, broadly construed, is absent or represented by reductionist images such as hamburgers and honey pots, what remains is individual pathology: bad eating and bad genes.

We acknowledge several limitations. First, we restricted our analysis to one medical journal, *JIM*. We selected this journal because of its role in a recent, high-profile controversy that exemplified the promises and pitfalls of graphical abstracts in biomedical publication, its high-impact status, and the presence of a ready gallery of graphical abstracts at the time of analysis. We recognize that our singular focus on *JIM* may mean that the biases we identified reflect those of select individuals, rather than a systemic issue in the publication of graphical abstracts in medicine. Future research should determine whether the patterns we observed here are more widespread, vary across journals, or are distinctive of *JIM*.

Second, we included graphical abstracts only through May 2020, when we began data collection and analysis. It is possible that controversy over the Gower and Fowler graphical abstract led to changes in editorial policies at *JIM* that may alter the patterns we observed here. Furthermore, graphical abstracts are optional for authors submitting to *JIM* and may reflect selection bias.

Third, we did not systematically analyze the textual abstracts or articles accompanying the graphical abstracts and were therefore unable to comment on whether graphical abstracts faithfully represented the article content for the sample as a whole. Our three case examples suggest that graphical abstracts in *JIM* do not always reflect the content of the article and may introduce assumptions or messages that the authors did not intend. However, we did not extend this analysis to all 140 abstracts. Future analyses should evaluate consistency of key messages and concepts across graphical abstracts, textual abstracts, and full articles.

Despite these limitations, our study raises new questions about how assumptions regarding race, genes, and disease manifest in graphical abstracts. Whereas other studies have examined the design of graphical abstracts^77^ and documented their prevalence and distribution across the social sciences,^3^ to our knowledge, our study is the first systematic analysis of the content of graphical abstracts in biomedical publication.

## Health Equity Implications

The gender, skin color, and size bias evident in graphical abstracts may contribute to misinformation, stigma, and mistreatment of women and gender nonconforming patients, black and brown patients, and larger bodied patients. These patients may experience delayed diagnoses and adverse clinical care experiences, in part, due to the negative messaging imbued in graphical abstracts of medical science. Furthermore, unwarranted emphasis on genetic rather than sociostructural contributors to health may further promulgate harmful racial essentialism and race-based medicine and hinder health policy reform. Visible inattention to sociopolitical and environmental factors implies the relative lack of importance for social support and policy interventions in improving population health. This message may detract from important policy interventions—such as education, nutrition, and health care access—even though they are often the most cost-effective and efficacious way to advance health equity.^78,79^

## Conclusion

Our results highlight the need for critical reflection on how to maximize the benefits of graphical abstracts while minimizing potential hazards. Previous researchers have noted potential pitfalls of graphical abstracts, including the risk of oversimplification, exacerbating biases, and quality control.^1^ Our work provides empirical evidence that these perils have manifested in at least one leading biomedical journal. Graphical abstracts in *JIM* include problematic representations of race, obesity, and disease that may increase prejudice in readers.^65–67^ Visual representations of bodies and disease in our sample are also skewed toward reductionist models of disease that stigmatize individual behavior, fail to account for social environments, and thereby reinforce false confidence in the magnitude of genetic contributors to health. In addition, the graphics lack diversity and are skewed to imagine the standard body as one that is slim, male, and white.

We recommend that medical journals develop standards for mitigating bias in the publication of graphical abstracts that (1) ensure diverse and inclusive representation of phenotype (e.g., skin tone) and gender; (2) mitigate weight bias; (3) avoid racial or ethnic essentialism; and (4) pay attention to sociostructural contributors to disease. As scholars at the Urban Institute have noted, empathically engaging and reflecting on the complex lived experiences of individuals represented by study data are critical to health equity. This includes attention to color, shape, iconography, order of data, missingness, context, purpose, and audience need.^80^ As scientists strive to reach broader audiences through graphical abstracts, such standards will optimize effective science communication while minimizing ethical harm.

## Supplementary Material

Supplemental data

## Abbreviations Used

BMI: body mass index
CI: confidence interval
CNTR: Chinese National Twin Registry
GWAS: genome-wide association studies
hsCRP: high-sensitivity C-reactive protein
*JIM*: *Journal of Internal Medicine*
