# Supplementary material for: Racial, Gender, and Size Bias in a Medical Graphical Abstract Gallery: A Content Analysis
Source: Health Equity. 2023 Sep 27;7(1):631–43. doi: 10.1089/heq.2023.0026 (PMC10541937; doi:10.1089/heq.2023.0026)
Supplement: Supplemental data [file Suppl_Data.docx]

**Appendix 1: Codebook**

**Codebook**

| 1 body |
| --- |
| 1.1 population |
| 1.1.1 demographic |
| 1.2 evolution |
| 1.3 silhouette |
| 1.3.1 skin tone |
| 1.4 illustration |
| 1.5 gender |
| 1.5.1 male |
| 1.5.2 female |
| 1.6 body size |
| 1.6.1 large body size |
| 1.7 life course |
| 1.7.1 aging |
| 1.7.2 intrauterine |
| 1.7.3 youth |
| 2 environment |
| 2.1 social |
| 2.2 lifestyle |
| 2.2.1 physical activity |
| 2.2.1.1 sedentary |
| 2.2.2 diet |
| 2.2.2.1 fast food |
| 2.2.2.2 fruits and vegetables |
| 2.2.3 alcohol |
| 2.2.4 smoking |
| 2.3 physical |
| 3 genetics |
| 3.1 genes |
| 3.2 epigenetics |
| 4 levels of organization |
| 4.1 organ+ |
| 4.2 sub-organ |
| 5 pathology |
| 5.1 neuropsych |
| 5.2 lung disease |
| 5.3 autoimmune |
| 5.4 endocrine |
| 5.4.1 diabetes |
| 5.5 cancer |
| 5.6 GI |
| 5.7 infection |
| 5.8 cardiovascular |
| 6 racialization |
| 6.1 origin |
| 6.1.1 map |
| 6.1.2 racial or ethnic category |
| 6.2 skin phenotype |
| 6.2.1 light |
| 6.2.2 dark |
| 7 intervention |

**1 body**

This category refers to visual representations of human bodies or descriptions of human bodies by size, sex or gender, age, etc.

**1.1 population**

This code is defined as a group of more than 5 human bodies and references to populations.

INCLUDES:

- "population"

NOTES: Codes for "population" and "silhouette"/"gender"/"skin phenotype" are mutually exclusive.

**1.1.1 demographic**

This code is used to refer to populations for which gender(s) or skin phenotype(s) are represented.

NOTE: "gender"/"skin phenotype - light/dark"/"silhouette" should not also be coded.

**1.2 evolution**

This code is used to refer to depictions of human evolution (i.e., apes, hominids, hominins, homo sapiens)

**1.3 silhouette**

This code is used to refer to representations of human bodies as silhouettes (i.e., without detail of physical features as if rendered in light).

**1.3.1 skin tone**

This code is used when silhouettes are shaded in skin tones, including light pinks, tans, and browns. If this code is applied, "light' or "dark" must also be coded.

**1.4 illustration**

This code is used to describe illustrations of human bodies (i.e., when physical features are rendered in recognizable detail as if shown under light).

INCLUDES:

- illustrations of bodies without skin (e.g., skeletons, vein systems)

**1.5 gender**

This category classifies representations of gender. This is also a code referring to mentions of sex or gender and also classifies male and female gender expressions of bodies. If gender is uncertain, no code is applied.

INCLUDES:

- "sex"

- "gender" / "pan-gender"

- intertwined male/female symbols

**1.5.1 male**

This code refers to a body gendered as man or male and the text "man," "men," or "male(s)." Signifiers include Mars symbol, male genitalia, short hair, Adam's apple, defined musculature, male restroom stick figure, etc.

**1.5.2 female**

This code refers to a body gendered as woman/female and the text "woman," "women," or "female(s)." Signifiers include Venus symbol, breasts, long hair, wide hips, gravid abdomen, dress-wearing, female restroom stick figure, etc.

**1.6 body size**

This code classifies visual and textual representations of bodies of different sizes, including references to "BMI" and "body weight."

INCLUDES:

- BMI

- body mass

- (body) weight

- scales

- body composition

- lean

- non-obese

**1.6.1 large body size**

This code refers to representations of obesity, overweight, and large or larger-than-average body size (i.e., depictions of abundant abdominal fat).

INCLUDES:

- BOTH visual and textual representations of large body size / obesity

- "overweight"

- "obesity"

- "increased BMI"

**1.7 life course**

This code includes representations of progression through the life course (i.e., from in utero through advanced age) and the various stages (i.e., older adult).

INCLUDES:

- intergenerational

**1.7.1 aging**

This code refers to the process of aging, senescence, or advancing into late life.

INCLUDES:

- "aging"

- visual representations of older adults (i.e., white hair, kyphotic spines, wrinkles)

- "elderly"

- "older"

**1.7.2 intrauterine**

This code is used to refer to depictions of the intrauterine environment (i.e., fetus within mother's womb).

INCLUDES:

- intrauterine

- womb

- intrauterine environment

- gestational

**1.7.3 youth**

This code refers to visual depictions of or textual references to infants, children, adolescents (i.e., smaller or youthful-looking bodies), or "younger" populations.

**2 environment**

This category is used to refer to visual representations of the environment (i.e., social, physical). This is also a code for textual references to "environment" or "environmental factors."

**2.1 social**

This code is used to refer to social dimensions or experiences as contributive variables to health outcomes.

INCLUDES:

- "stigma"

- "low education"

- "high income"

- "living alone"

- psychosocial

- "stress"

- "perceptions"

**2.2 lifestyle**

This category is used to refer to individual lifestyle or choices that affect health outcomes.

INCLUDES:

- "lifestyle"

- "shift work"

- "sleep"

- "jet lag"

- "chronotype"

- "circadian"

- "pregnancies"

**2.2.1 physical activity**

This code is used to refer to physical activity or an active lifestyle.

INCLUDES:

- bodies in motion

- sports

- other physical activity

- "mobility"

- "fitness"

**2.2.1.1 sedentary**

This code is used to refer to low physical activity or sedentary lifestyle.

INCLUDES:

- "sedentary"

- "low physical activity"

- sleeping bear

- body working at computer

**2.2.2 diet**

This category refers to visual or textual representations of diet, including food and beverages.

INCLUDES:

- omega-3 fatty acids

- fish

- cooking/fire

- fruits and vegetables

- "diet"/"dietary"

- "foodome" / "food availability"

- vitamins

**2.2.2.1 fast food**

This code is used for depictions of fast food (i.e., french fries, hamburgers, soda).

**2.2.2.2 fruits and vegetables**

This code is used for visual representations or textual references to fruits and vegetables.

**2.2.3 alcohol**

This code is used to refer to depictions or references to alcohol (e.g., beer, wine, spirits).

**2.2.4 smoking**

This code is used for visual representations of or textual references to cigarettes or cigarette smoking.

**2.3 physical**

This code encompasses referencesto the physical environment.

INCLUDES:

- "exposome"

- lightning bolts

- temperature

- UV radiation / sunlight / light pollution

- pain-causing substances

- PCBs and environmental contaminants

**3 genetics**

This code category is used to subdivide genetics/DNA and epigenetics or gene expression.

**3.1 genes**

This code is used to refer to representations of human genes and DNA.

INCLUDES:

- genes

- genetic mutations

- gene expression

- risk alleles

- chromosomes

- gene editing

- genome

- gene replacement

- DNA and DNA helices

- mitochondrial DNA

- candidate genes

EXCLUDES:

- epigenetics

- "gene expression"

- epigenome

- bacterial and viral DNA

**3.2 epigenetics**

This code refers to mentions of epigenetics or gene expression (vs. coding DNA); this suggests that gene expression can be modulated by internal or external inputs via signal transduction.

INCLUDES:

- "epigenetics"

- "gene expression" ONLY IF contextualized in response to an exposure

- histone modification

- methylation or acetylation

- "phenotype"

- RNA interference (e.g., tsRNA, miRNA)

- microbiome

**4 levels of organization**

This category is used to describe pathology within various levels of organization within the human body, including organ systems, organs/tissues, cells, organelles, and molecules/macromolecules.

EXCLUDES:

- textual representations of pathology (only visual representations should be coded)

**4.1 organ+**

This code refers to pathology occurring at the level of the organ or greater (i.e., organ or organ system). The pathology should occur at a level larger than a tissue or cell.

INCLUDES:

- organs + connective tissue

- multiple connected organs

- aorta

- teeth/gums

- heart

- brain

- lungs

- skin

- a network of vessels

EXCLUDES:

- an isolated vessel or a cross-section of a vessel

- any pathology at a level smaller than a tissue or vessel

**4.2 sub-organ**

This code refers to pathology occurring at the level BELOW an organ, including tissues, cells, organelles, or molecules.

INCLUDES:

- isolated vessel or cross-section of vessel

- bacterial cells

- adipose tissue

- immune cells

- nuclei

- mitochondria

- antibodies

- viral particles

**5 pathology**

This category is used to classify representations and textual references to types of disease or aberrations in normal physiological function. Unless otherwise specified (as "healthy," "normal," "resting"/"homeostatic," "physiologic," "simulation"/"experimental," etc.) inclusion in an abstract will presuppose pathology.

This category will also be used to code diseases or pathologies not otherwise specified (e.g., "disease," "dysfunction," "unhealthy" as isolated terms and pathology arising from other organ systems).

Code INCLUDES:

- musculoskeletal disease (e.g., "fracture" or broken bones)

- hematologic disorders (e.g., "iron deficiency anemia")

- mitochondrial disease

- dermatology (e.g., scar)

- sleep disorders

- periodontal disease

- osteopenia / osteoporosis

- "frailty"

**5.1 neuropsych**

This code is used to refer to pathology involving the neurologic or psychiatric systems, including dementia and depression.

INCLUDES:

- neurodegeneration

- brain atrophy

- suicide

- pain

- neurological conditions caused my mitochondrial aberrations

- Parkinson's disease

- Alzheimer's disease / dementia

- hearing loss / deafness

- blindness

- neurotransmission

- depression

- muscle disease / myopathy

- Leigh syndrome

- fatal mitochondrial encephalopathy

EXCLUDES:

- cancers involving the nervous system

- autoimmune disorders involving the nervous system

**5.2 lung disease**

This code is used to refer to pathology involving the lungs, including COPD.

INCLUDES:

- hypoxia

- obstructive sleep apnea

- COPD

EXCLUDES:

- COVID or other lung infection (e.g., pneumonia --> should be classified as "infection")

- autoimmune disorders affecting the lungs

**5.3 autoimmune**

This code refers to autoimmune or inflammatory conditions or visual representations of autoimmune pathologies.

INCLUDES:

- antimitochondrial-antibody / AMA positive

- inflammatory (as in, "inflammatory (gene) response")

- Sjogren's syndrome

- "immune-related"

- HLA

- IG4-RD

- granulomatous inflammation

- angioedema

- rheumatoid arthritis

EXCLUDES:

- infection

- "immunity" as an isolated term

**5.4 endocrine**

This code is used to refer to pathology involving the endocrine system or components of the endocrine system, including Cushing's disease. Diabetes is a subcode.

INCLUDES:

- hormone dysfunction

- adipose tissue

- Cushing's disease

- hypercortisolism / increased cortisol / cortisol molecule

- familial chylomicronemia syndrome

- lipid metabolism / hyperlipidemia

- testosterone / "testosterone levels"

- Vitamin D and PTH

- "metabolic morbidity"

- "energetic insufficiency"

EXCLUDES:

- normal glucose metabolism

**5.4.1 diabetes**

This code is used for textual references to ALL types of diabetes,

INCLUDES:

- type 1 and type 2 diabetes

- gestational diabetes

- prediabetes

- hyperglycemia

- HbA1c >5.5% / glucometer

**5.5 cancer**

This code is used to refer to cancer, including tumors and tumor cells. Cancer supersedes any involved organ system (e.g., colorectal cancer would be coded as "cancer", NOT "GI".)

INCLUDES:

- lymphomas (e.g., DLBCL, Hodgkin's)

- chemotherapies (e.g., "RCHOP")

- melanoma

- MDS (myelodysplastic syndromes)

- myeloproliferative neoplasms

- tumor

- tumor biomarkers

**5.6 GI**

This code includes gastrointestinal disease such as IBS.

INCLUDES:

- pancreatitis

- primary biliary cholangitis (PBC)

- GI disease caused by mitochondrial disease

- Crohn's disease

- fatty liver / NAFLD

- irritable bowel syndrome (IBS)

- biliary atresia

- liver failure

- enteropathy

**5.7 infection**

This code is used to refer to infections or infection-causing agents (i.e., pathogens such as viruses or bacteria).

INCLUDES:

- "infection"

- bacterial cells

- viral molecules / "virus"

- bateremia

- influenza / flu

- pneumonia

- HIV / HIV ribbon

- "transmission risk"

- "sepsis"

- HPV

- Hepatitis C

- COVID-19

- Lemierre syndrome

**5.8 cardiovascular**

This code is used to refer to pathology affecting the cardiovascular system, including the renal system.

INCLUDES:

- cardiomyopathy

- atherosclerosis

- coronary artery disease

- "heart disease"

- hyperlipidemia

- stroke

- Barth syndrome

- congenital heart defects

- hypertension

- atrial fibrillation

- uremic media calcification

- myocardial infarction

- postural tachycardia syndrome

- chronic kidney disease

- abdominal aortic aneurysm

- thromboembolism

**6 racialization**

This code is used for markers of origin or racial identification, including skin color phenotype

**6.1 origin**

This code is used when mention of the origin of the population studied is referenced. Origin refers to continent, nationality, race, or ethnicity, nationality. If multiple origins are included in an abstract, each should be coded separately.

**6.1.1 map**

This code is used to refer to maps or globes; should be coded separately from "origin."

**6.1.2 racial or ethnic category**

This code refers to descriptions of racial or ethnic groups (e.g., White, Black, Hispanic). If multiple racial or ethnic categories are referenced in an abstract, each should be coded separately.

INCLUDES:

- ethnicity

- race

- Black / African American

- White / Caucasia

- Hispanic / Latino/a/x

- Asian

**6.2 skin phenotype**

This category is used for visual representations of human bodies of different skin tones. If multiple skin phenotypes are present in an illustration, both "light" and "dark" may be applied.

1-5 = light

6+ = dark

https://perla.soc.ucsb.edu/data/color-palette

**6.2.1 light**

This code used for visual representations of human bodies with light skin tones

1-5 = light

https://perla.soc.ucsb.edu/data/color-palette

**6.2.2 dark**

This code used for visual representations of human bodies with dark skin tones

6+ = dark

https://perla.soc.ucsb.edu/data/color-palette

**7 intervention**

This code is used to refer to the clinical setting and interventions, including injections, surgeries, or drugs.

INCLUDES:

- surgery

- injections

- vaccines

- drugs/medicines/opioids

- "therapy"

- stethoscope

- hospital

- hospital bed

- diagnostic procedures (e.g., EKG, tubes of blood)

- "adherence"

- NORSWITCH

- UK BioBank
